# Weight Loss and Gastrointestinal Hormone Variation Caused by Gastric Artery Embolization: An Updated Analysis Study

**DOI:** 10.3389/fendo.2022.844724

**Published:** 2022-03-16

**Authors:** Yi Tang, Xiaohui Pan, Ge Peng, Nanwei Tong

**Affiliations:** ^1^Department of Endocrinology and Metabolism, West China Hospital of Sichuan University, Chengdu, China; ^2^Laboratory of Diabetes and Islet Transplantation Research, Center for Diabetes and Metabolism Research, West China Hospital of Sichuan University, Chengdu, China

**Keywords:** gastric artery embolization, obesity, weight loss, gastrointestinal hormone, ghrelin

## Abstract

**Objective:**

Many gastric artery embolizations (GAE) have been performed in recent years. We try to determine whether GAE caused weight loss by decreasing gastrointestinal hormone through the analysis of weight loss and gastrointestinal hormones changes.

**Methods:**

The PubMed and Medline databases, and the Cochrane Library, were searched using the following keywords. A total of 10 animal trials (n=144), 15 human trials (n=270) were included for analysis. After GAE, we mainly evaluated the changes in body weight loss (BWL) and body mass index (BMI), as well as metabolic indexes, such as blood glucose, lipids, and gastrointestinal hormones levels.

**Results:**

Animal subjects received either chemical or particle embolization, while human subjects only received particle embolization. In animal trials (growing period), the GAE group gained weight significantly slower than the sham-operated group, ghrelin levels decreased. In human trials, GAE brought more weight loss in the early stages, with a trend towards weight recovery after several months that was still lower than baseline levels. Besides weight loss, abnormal metabolic indicators, such as blood glucose and lipids were modified, and the quality of life (QOL) scores of obese patients improved. In addition, weight loss positively correlates with ghrelin.

**Conclusion:**

GAE may help people lose weight and become a new minimally invasive and effective surgery for the treatment of modest obesity. Physiologic changes in gastrointestinal tract of gastrointestinal hormones level may be one reason for weight loss in GAE.

## Introduction

Obesity has become a major chronic disease in recent years, with an increasing number of people suffering from it. Obesity-induced insulin resistance is the key factor of the metabolic syndrome, which can lead to metabolic diseases, such as type 2 diabetes (T2D) and hypertension, as well as sleep apnea, cardiovascular, musculoskeletal, reproductive, and psychological disorders (1). More weight loss within a certain range leads to greater benefits and obesity therapy is a critical health initiative.

Weight loss treatment includes lifestyle modification (diet and exercise), drug, and surgery. Lifestyle intervention and drug sometimes fail to reduce weight, especially in severely obese patients (2). Metabolic surgery is a highly effective intervention for metabolic syndrome that can promote substantial weight loss (3). Every year, 685,000 metabolic surgeries are performed worldwide. The three most commonly performed procedures are the Roux-en-Y gastric bypass (RYGB), sleeve gastrectomy (SG), and one-anastomosis gastric bypass (OAGB) (4, 5). The changes in gastrointestinal anatomical structure during weight loss surgery lead to neural and physiological changes that affect hypothalamic signals, intestinal hormones, bile acids, and intestinal microbiota. These changes limit feeding behavior and nutrient absorption to achieve weight loss. RYGB and SG result in the postprandial secretion of glucagon-like peptide-1 (GLP-1), peptide YY (PYY), and oxyntomodulin (OXM), which can increase satiety (6). These hormones regulate glucose homeostasis by altering insulin secretion and sensitivity in the early postoperative period (7). Laparoscopic technology is improving, and the incidence of postoperative complications (including gastric perforation, intestinal obstruction, mucosal tears, and gastrointestinal bleeding) are decreasing. However, still a significant number of patients rely solely on lifestyle intervention and drug due to their fear of surgery, resulting in weight loss failure (8). GAE is a new, minimally invasive surgery in which a physician inserts an angiographic catheter into the groin or wrist, navigates to the aorta, upper abdominal aorta, and left gastric artery through the femoral artery or radial artery. Following that, embolization is performed and the catheter is removed; this is known as transarterial embolization of the gastric fundus (9). Because the gastric fundus is mainly supplied by the left gastric artery (LGA) and a small part by the gastroepiploic artery, embolization has been performed primarily on the left gastric artery in most studies (10). This review describes animal and clinical trials to analyze the therapeutic effects and potential mechanisms of GAE.

## Methods

**Figure 1:**
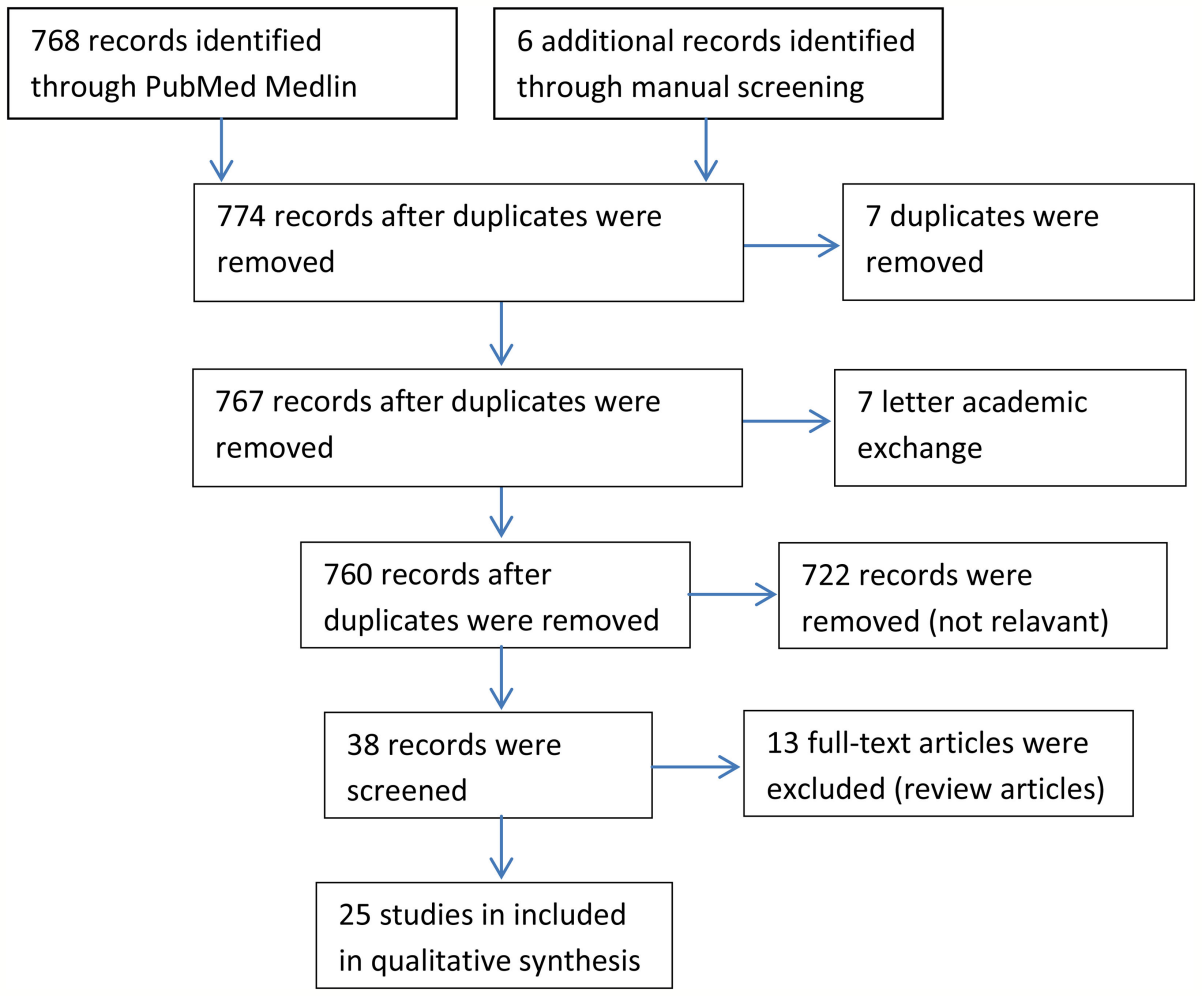
Flow diagram of study selection process.

We searched out 768 articles by using gastric artery embolization as a keyword which included 3 meta-analyses (11–13), 7 systematic reviews (14–20), 3 systematic reviews only analysed embolic material choices or procedures (21–23), 15 articles about materials, 722 articles analyzed other indexes just like hepatocellular carcinoma, 7 academic discussions, and 7 duplicates (**Figure 1**). Finally, we counted 25 articles involving changes in body weight after GAE (10 animal trials [n=144 (24–33), 15 human trials (n=270) (33–48)] different from previous reviews.

## Results: The Efficacy of Gastric Artery Embolization

### Weight Loss

#### Animal Experiments

In all animal experiments, control groups were injected with normal saline (**Table 1**). Arepally et al. (24) initially reported that GAE could slow down the natural weight gain of developing pigs with a low dose of morrhuate sodium. In the following year, they (25) found that injecting a standard dose of morrhuate sodium into the left gastric artery supplying the gastric fundus significantly delayed weight gain in the experimental group compared to the control group. Bawudun et al. (26) used hydrochloride/lipiodol (group A) and polyvinyl alcohol (PVA) particles (group B) in different groups and discovered body weight loss in groups A and B, in contrast to the sham group. Furthermore, subcutaneous fat was significantly reduced in both groups (P<0.5). Later, solid substances were used in the following seven animal experiments (4-12 weeks) (27–33). Although the animals, embolic agents, embolic sites, and follow-up times used in the above studies were not identical, it was shown that GAE either prevented weight gain in developing animals or reduced weight in adult animals. However, GAE cannot achieve equal weight loss as SG.

**Table 1:** Body weight and GI hormones changes after GAE in different animals.

Study reference	Subject	FU weeks	Design and group intervention(n),control(n)	Embolic agent(dose or size)	Weight(kg or g)		P value (intervention: control)	Ghrelinbasic/change% (P value)intervention vs control	Leptinbasic/post-GAE or change%(P value,intervention vs control)	Adverse events(%)
					intervention basic/change%	control basic/change%				
Arepally	swine	4	GAE(6),sham(2)	morrhuate sodium	40-45kg/+1.4%	40-45kg/+8.6%	*P*>0.05	---	---	ulcer100%
2007 (24)				(37.5-62.5μg)						
Arepally	swine	4	LGAE(5,group A).	morrhuate sodium	40-45kg/+7.8%	40-45kg/+15.1%	*P*<0.05	1104 ±74.8pg/mL/-34%,-38.6%,-42.5%,-12.9%(1-	---	---
2008 (25)			sham(5,group B)	(125μg)				,2-,3-,4- weeks,group A).1078±161.9pg/mL/-		
								1.7%,-9.7%, +2.6%, +18.2%(1-,2-,3-,4-		
								weeks,group B).(*P*<0.02,<0.001,<0.001,<		
								0.03,A vs B,1-,2-,3-,4- weeks,respectively).		
Bawudun	dog	8	LGAE(5,group A).	bleomycin A5 hydrochloride and	14.8±0.3kg/↓	13.9±0.9kg/↑	*P*=0.000(A vs C).	1424.7±94.6pg/mL/-15.8%(group A).	---	0
2012 (26)			LGAE(5,group B).	lipiodol(group A).	(group A).	(group C)	*P*=0.000(B vs C)	1071.2±86.4pg/mL/-30.16%(group B).		
			sham(5,group C)	PVA particles(group B)	15.3±0.9kg/↓			1180.1±132.5pg/mL/+13.6%(group C).		
					(group B)			(*P*=0.007,A vs C),(*P* =0.004,B vs C)		
Paxton	swine	8	GAE(6,group A).	calibrated microspheres(40μm)	39.4±4.9kg/+9.1%	37.4±5.3kg/+25.1%	*P*=0.025	1605.7±211.4pg/dL/-33.5%(group A).	---	ulcer40%
2013 (27)			sham(6,group B)					1591.6±215.3pg/dL/+20.6%(group B).		
								(*P*=0.004, A vs B)		
Pasciak	swine	9	LGAE(6,group A).	90Y resin microspheres	21.8-28.1kg/+61.1%	21.8-28.1kg/+119%	*P*=0.053	-/↓(group A)	---	ulcer83.3%
2016 (28)			sham(2,group B)	(46.3-105.1MBq)						
Kim	swine	5	GAE(5,group A).	non-spherical PVA particles	31.8±5.8kg/+29.6%	35.1±9.5kg/+32.7%	*P*>0.05	880.0±559.5pg/mL/-41.5%(group A)	---	ulcer60%
2017 (29)			sham(5,group B)	(150-250 or 50-150μm)						
Legner	rat	12	GAE curvature(8,group A).	a bulking agent	469±32.3g/+32.2%	469±32.3g/+32.6%	*P*=0.038(A vs B).	-/↓ (three groups)	-/15.2 ±3.8ng/ml(group A).	---
2020 (30)			GAE cardia(8,group B).		(group A)	(group C)	*P*=0.01(B vs C)	(*P*>0.05, AvsC and BvsC)	-/10.48±3ng/ml(group B).	
			sham(6,group C)		469±32.3g/+26.4%				-/16.2 ±4.6ng/ml(group C).	
					(group B)				(*P*= 0.01,A vs B,*P*=0.008,B vs C)	
Liu	pig	12	LGAE(8,group A).	PVA I36particles(500μm)	12.54±0.91kg/-15.4%	12.45±0.89kg/+12.1%	*P*<0.001(A vs D, B vs D).	1429.34±121.37pg/ml/-16.6%(group A).	5.87±0.93µg/L/-53.7%(group A).	ulcer12.5%
2021 (31)			RGAE(8,group B).		(group A)	(group C)	*P*<0.01 (A vs C, B vs C).	1428.7 4±119.47pg/ml/-11.3%(group B).	5.94±0.78µg/L/-47.9%(group B).	gastritis25%
			sham(8,group C). healthy(8,group D)		12.61±0.97kg/-13.5% (group B)	9.52±0.53kg/+5.2% (group D)	*P*>0.05(A vs B)	1413.63±116.21pg/ml/-3.2%(group C). 1102.23±95.34 pg/ml /+2.6%(group D).	5.92±0.96µg/L/+1.7%(group C). 2.31±0.42 µg/L/+4.3%(group D).	
								(*P*<0.01,A vs C,C vs D)(*P*>0.05,A vs B vs D)	(*P*>0.05,A vs C,B vs C,D vs C,P<0.05,A vs B vs D)	
Yardimci	rat	4	LGAE(5,group A).	5.0 polyglycolic acid suture	432.4±61.4g/-0.1%	423.6±35.1g/-	---	67.32±81.7pg/ml/-41.6%(group A).	387.6±272.2pg/ml/+89.3%(A).	---
2017 (32)			SG(5,group B).		(group A)	2.1%(group C)		65.72±54.2pg/ml/-16.9%(group B).	449.1±149.1pg/ml/+29.9%(B).	
			sham(5,group C)		418±69.3g/-24.1%			45.6±32.7pg/ml/+10.5%(group C).	187.7±106.2pg/ml /+278%(C)	
					(group B)			(*P*=0.9,A vs B)	(*P*=0.3,A vs B vs C)	
Diana	pig	4	GAE(7)(group A).	microspheres-α(500-700μm)(group A).	---	---	---	1642±545.8pg/mL/-47.3%(group A).		ulcer62.5%
2015 (33)			GAE(5)(group B)	microspheres-b(100-300μm)(group B)				1564±359.9pg/mL/-64.1%(group B)		

GI: gastrointestinal, GAE: gastric artery embolization, LGAE: left gastric artery embolization, RGAE: right gastric artery embolization, SG: sleeve gastrectomy, FU: follow-up, PVA: polyvinyl alcohol, Errorbars=standard errors of the mean.

#### Human Trials

**Figure 2:**
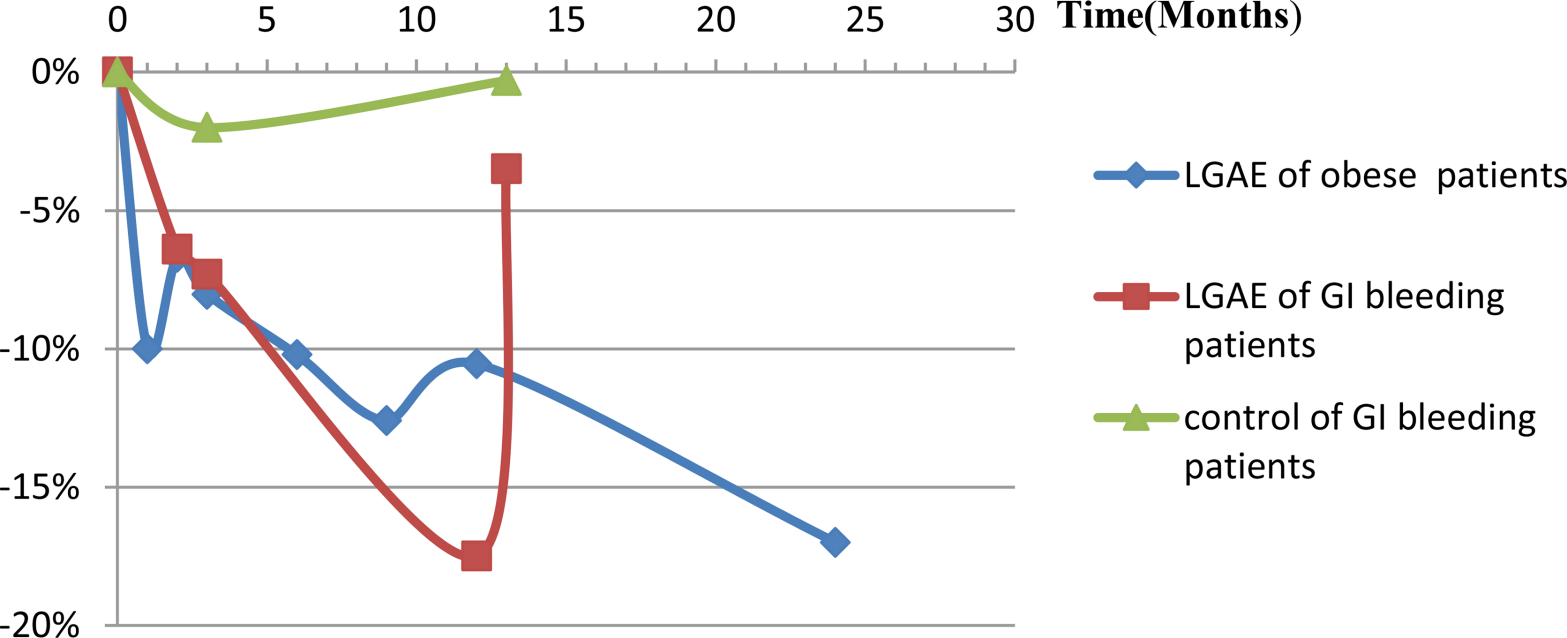
Body weight loss (%) changes post-LGAE in GI bleeding or obese patients. LGAE, left gastric artery embolization; GI, gastrointestinal.

Originally, two retrospective cohort studies (34, 36) compared fundic artery embolization with other arterial embolization for GI bleeding and showed significantly more body weight loss after GAE (*P*=0.006, 0.18, at 3-, 13-months, respectively). According to Gunn et al., weight recovery after 13 months was due to the short duration of weight loss, which may be related to revascularization or collateral artery formation after LGAE. In the experimental group, 58% had a history of malignancy and four patients were receiving chemotherapy during the study period, while 50% of the control group had a history of malignancy and six patients were receiving chemotherapy during the study period. Although there was no significant difference in the proportion of patients with a history of malignancy (*P*=0.29) or receiving chemotherapy (*P*=0.97) between the two groups, there was still an effect on the results. Then, two similar LGAE studies calculated patients without tumor for GI bleeding (41, 44), also found body weight loss, decreased BMI, excess body weight (EBW, basic 23.3 ± 10.6kg, -24.1%, P=0.003), and body fat index (BFI, basic 128.6 ± 54.6cm^2^/m^2^, -3.7%, P=0.03), subcutaneous fat index (SFI, basic 81.7 ± 44.5cm^2^/m^2^, -4.1%, P=0.03), and skeletal muscle index (SMI, basic 44.5 ± 7.2cm^2^/m^2^, -6.9%, P<0.001), but not visceral fat index (VFI, basic 35.8 ± 17.8cm^2^/m^2^, -4.1%, P=0.13) and intramuscular fat index (IMFI, basic 10.2 ± 4.8cm^2^/m^2^, -1.6%, P=0.83). Therefore, the latter eleven prospective studies with self-control design only included simple obese patients were conducted. GAE caused greater weight loss in the early stages, with a trend towards weight recovery after several months that was still lower than baseline levels (*P*<0.05) (**Figure 2**). Besides the observed weight changes, Levigard et al. (48) showed that both BMI and waist circumference (WC) decreased (basic 108.6 ± 11.16cm, -5.5%, P=0.03). Except WC decreased (basic 114.34 ± 10.40, -10.8%, P=0.122), waist-to-hip ratio(WHtR) decreased (basic 69.99 ± 4.94%, -10.8%, P=0.125), Bai et al. (40) further discovered a significant reduction in abdominal adipose tissue, including the area of subcutaneous adipose tissue (SAT,basic 400.90 ± 79.25cm^2^, -20.09%, -18.11%, -28.52%, P= 0.006, 0.02, 0.101, at 3-, 6-, 9-months, respectively), and total abdominal adipose tissue (TAAT,basic 695.8 ± 107.43cm^2^/-9.18%, -11.26%, -24.29%, P=0.169, 0.006, 0.107, at 3-, 6-, 9-months, respectively), using MRI (**Table 2**).

**Table 2:** Body weight and GI hormones changes post-GAE in GI bleeding patients (retrospective cohort studies and self-control study) and obese patients (self-control studies).

Study reference	Subject	FU months	Design and group		Embolic agent/n embolic(size)	Weight(kg) basic/change% (P value, intervention vs control or self-control)	BMI(kg/m^2^) basic/change(%) (P value, intervention vs control or self-control)	Metabolic variablesbasic/post-operation (P value, self-control)	Gastrointestinal hormones:basic/change% (P value, self-control)	Adverse events(%)
			intervention(n), control(n) retrospective	intervention(n) self-control						
Gunn 2014 (34)	GI bleeding patients	13	LGAE(19,group A) control(splenic arteries13, gastroduodenal arterie7, right hepatic arterie4,left hepatic arterie4,group B)		coils/9,gelfoam/5,PVA particles/5(group A). coils/23,gelfoam/3, PVAparticles/2 (group B). PVA particles(300-500μm, 500-710μm,710-1000μm)	-/-7.3%,-3.5%(3-,13-month,group A). -/-2%,-0.3%(3-,13-month,group B). (*P*=0.006,0.18,A vs B,3-,13-months,respectively),	30.3/-(group A). 29.2/-(group B).	---	**---**	---
Anton 2015 (36)	GI bleeding patients	12	LGAE(10,group A). control(gastroduodenal arter9, right hepatic artery1, pancreaticoduodenal artery2, right gastroepiploic artery1, branches of the cecal artery1, inferior pancreaticoduodenal artery1,inferior mesenteric artery3,right colic artery3, middle colic artery1,group B)		microcoils/5,gelatin sponge/3,coils and gelatin sponge/2(group A). microcoils/17,gelatin sponge/3,coils and gelatin sponge/1(group B). embospheres(700-900,900-1200μm)	214.6/-(group A). 181.0/-(group B).	42.2±6.8/-9.8%,-11.7%,-8.6%,-5%(1-,4-,8-,12-months,group A). 42.2±6.8/-4%,+0.1%,-1.7%,+2.6%(1-,4-,8-,12-months,group B). (*P*=0.042,0.033,0.32,0.42, A vs B,1-,4-,8-,12-months,respectively)	---	---	---
Kim 2018 (41)	GI bleeding patients	12	LGAE(39,21 patients visited at the end). no other embolization		coils/6,gelfoam slurry/19, PVA particles/5, combination embolics/9, PVA particles(100–300,300–500,500–700µm)	93.4/-17.5% (*P*=0.045,self-control)	29.9/-9.4% (*P*=0.045)	---	---	ulcer5.1%
Takahashi 2019 (44)	GI bleeding patients	1.5	LGAE(16). no other embolization		PVA particles	87.9±12.5/-6.4% (*P*=0.03,self-control)	30.0±4.3/-6.3% (*P*=0.005)	---	---	---
Kipshidze 2015 (35)	obese patients	24		LGAE(5)	compressible microspheres (300-500μm)	128±24/-10%,-13%,- 16%,-17%,-17%. (*P*=0.043,0.042,0.042,0.041,0.041, self-control,1-,3-,6-,12-,24-months,respectively)	42.2±6.8/-	---	ghrelin:471±21pg/mL/-29%,-36%,-19%,-21.9%(*P*=0.043,0.043,0.043,0.068,1-,3-,6-,12-months,respectively).	0
Syed 2016 (37)	obese patients	6		LGAE(5)	PVA particles (300-500μm)	118/-8.5% (*P*=0.0775,self-control)	42.4(40.2–44.9)/-	HbA_1c_ 7.4%/6.3%(*P*=0.051).	ghrelin:612/+5.3%pg/mL, leptin:basic 26ng/mL/-24%(6-months). CCK:basic44.5pg/mL/-25.8%(6-months)	ulcer60%
Weiss 2017 (38)	obese patients	3		LGAE(5)	PVA particles (300-500μm)	127.8±19.8/-3.7%	43.8±2.9/-	---	GLP-1:+106.6%(1-month). PYY:+17.8%(1-month)	ulcer20% subclinical pancreatitis20%
Weiss 2019 (39)	obese patients	12		LGAE(20)	PVA particles **(**300-500μm)	139±20/-5.5% (*P*=0.006,self-control)	45±4.1/-	HbA_1c_5.9%±0.4%/5.8%±0.4%,5.7%±0.5%(6-,12-months, *P*=0.047).FBG -8.5mg/dl(*P*=0.11). TG↓(*P*=0.06,0.45,0.30,0.90,1-,3-,6-,12-months,respectively). TC↓ (*P*=0.04,*P*=0.08,at 3-,12-months).LDL-C↓,12-months (*P*=0.08).HDL-C↑(*P*=0.03,0.89,0.10,0.006,1-, 3-, 6-,12-months,respectively).	---	ulcer10% subclinical pancreatitis5%
Bai 2018 (40)	obese patients	9		LGAE(5)	PVA particles (500–710μm)	102.0±16.19/-7.4%,-10.2%,-12.6% (*P*= 0.074,0.0479, 0.121,self-control, 3-,6-,9-months,respectively)	38.1±3.8/-	---	ghrelin:310.4± 95.79pg/ml/-40.83%,-31.94%,-24.82% (*P*=0.009,0.107,0.151,3-,6-,9-months,respectively). leptin:basic15.7± 6.6ng/mL/-0.26%,-4.33%,-11.22%(*P*= 0.929,0.427,0.295,3-,6-,9-months,respectively)	ulcer20%
Pirlet 2019-20 (42, 43)	obese patients	12		LGAE(7)	PVA particles (300-500μm)	160±27/-6.7%,-10.1%,-7.4%,2-,6-, 12- months,respectively	---	---	---	epigastric discomfort
MMA 2019 (45)	obese patients	6		LGAE(10)	PVA particles (300-500μm)	107.4±12.8/-8.9% (*P*<0.001,self-control)	37.4±3.3/-8.8% (*P*<0.001)	HbA_1c_6.1±0.2%/4.7±0.6%(-21.4%)(*P*<0.0001)	---	epigastric discomfort70%
Elens 2019 (46)	obese patients	12		LGAE(16)	PVA particles (500–700μm)	79±10/-12.3%	28.9±2.5/-11.7%	---	---	---
Reddy 2020 (47)	obese patients	12		LGAE(44)	PVA particles (300-500μm)	160/-8.1% (*P*<0.05,self-control)	39.6±3.8/↓			---
Levigard 2021 (48)	obese patients	6		LGAE(19)	PVA particles (300-500μm)	94.30±7.21/-6.8% (*P*=0.01,self-control)	36.37±2.58/-7%	HbA_1c_6.58%±1.72/4.74%±2.58(*P*=0.06). FBG111.10±47.80mg/dl/83.70±6.98mg/dl(*P*=0.01). Fasting insulin26.24±14.61lUI/ml/17.82±8.70lUI/ml(*P*=0.01). HOMAIR7.29±5.66/3.73±1.99(*P*=0.01). TG110 ±43.68 mg/dl/96.80±50.19 mg/dl(*P*=0.07). LDL-C 106.7±30.20mg/dl/92.70±24.90mg/dl(*P*=0.08); HDL-C 42.70±10.99mg/dL/42.80±9.46mg/dl(*P*=0.24).	---	ulcer10.5%

GI: gastrointestinal, GAE: gastric artery embolization, LGAE: left gastric artery embolization, FU: follow-up, BMI: body mass index, PVA: polyvinyl alcohol, HbA_1_c: glycated hemoglobin, FBG: free blood glucose,

TG: triglyceride, TC: cholesterol, LDL-C: low density lipoprotein cholesterol, HDL-C: high density lipoprotein cholesterol, HOMA-IR: insulin resistance index, CCK: cholecystokinin, GLP-1: glucagon-like peptide-1,

PYY: peptide YY, Errorbars=standard errors of the mean.

### Metabolic Indexes

**Figure 3:**
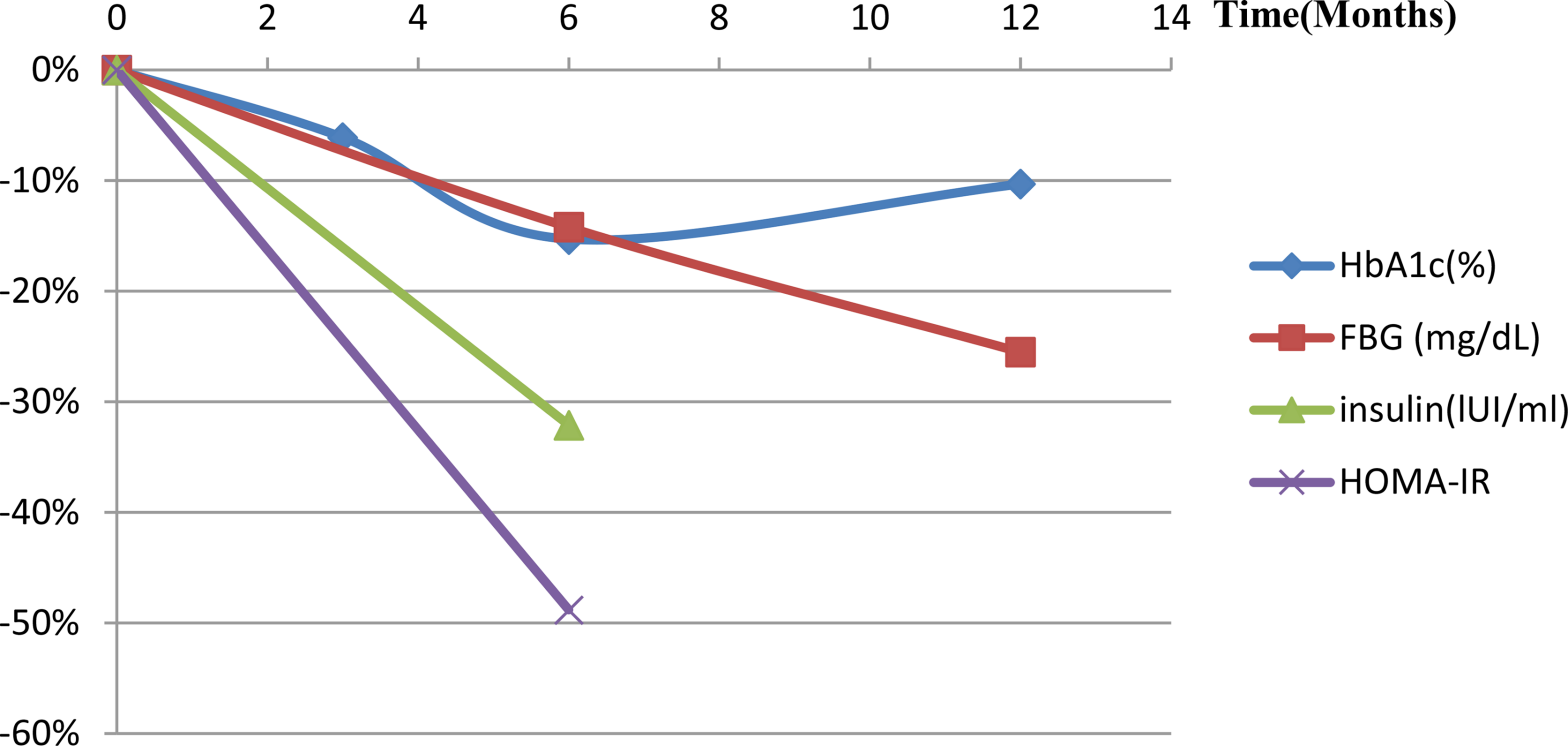
Metabolic index changes post-LGAE in obese patients. LGAE, left gastric artery embolization; HbA_1_c, glycated hemoglobin; FBG, free blood glucose; HOMA-IR, insulin resistance index.

**Figure 4:**
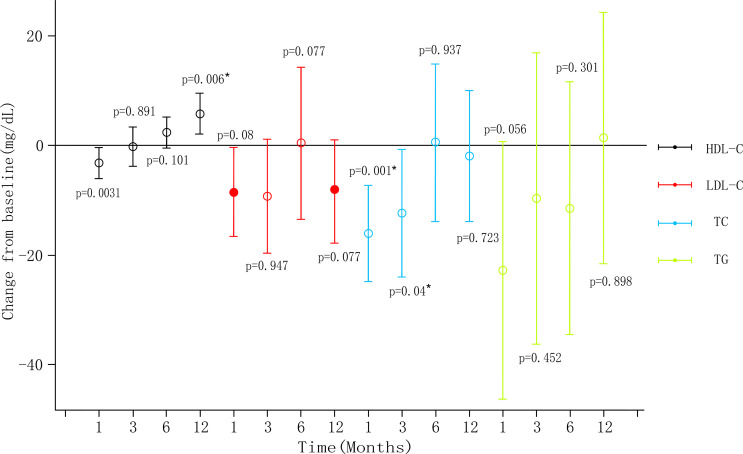
Blood lipid level changes post-LGAE in obese patients. LGAE, left gastric artery embolization; * indicates statistical significance, apllyied from weiss (39).

Only one animal trial (27) found no changes in serum glucose levels (*P*=0.81). Four human trials examined metabolic indicators (**Table 2**). Syed et al. (37) reported that one diabetic patient lost weight and HbA_1C_ level dropped from three to six months. Zaitoun et al. (45) showed HbA_1c_ reduced and there was a statistically significant positive correlation between reduced BMI and HbA_1_c (r=0.91, *P*=0.0002). Levigard et al. (48) found a decrease in fasting blood glucose (FBG), fasting insulin, insulin resistance index (HOMA-IR) (*P*=0.01), and HbA_1c_ levels (*P*=0.06) after six months without medication change (**Figure 3**). Furthermore, the serum triglyceride (TG) and low-density lipoprotein cholesterol (LDL-C) levels decreased (*P*>0.05), while high-density lipoprotein cholesterol (HDL-C) levels increased (*P*>0.05). Weiss et al. (38, 39) discovered that HbA_1c_ and blood glucose decreased at 12 months, while HbA_1c_ change did not correlate with weight change (r^2^ = 0.24). TG initially decreased but later rebounded to baseline level, total cholesterol (TC) and LDL-C levels decreased at 12 months, with the percentage of weight changes negatively correlating with LDL-C changes after 12 months (r^2^ = 0.24). HDL decreased one month after embolization but increased at all subsequent time (**Figure 4**). This suggests that GAE may improve metabolic indexes, but it is not clear whether it works by reducing body weight.

### Gastrointestinal Hormones

#### Animal Experiments

Serum ghrelin was evaluated in nine animal trials. According to Arepally et al. (24), ghrelin increased in the low-dose group but decreased in the high-dose group. This may be due to incomplete embolization of the gastric fundic mucosa in the low-dose group, which did not effectively inhibit ghrelin. Therefore, a standard-dose test was performed the following year (25), where ghrelin levels decreased in the GAE swine after one week. Following that, several other tests revealed significant ghrelin reductions in the LGAE (33). Bawudun et al. (34) reported that the mean post-procedure relative percentage of ghrelin was decreased, while ghrelin was increased in the control group. Compared to the baseline, the changes in subcutaneous fat in all animals positively correlated with the changes in ghrelin (r=0.632, *P*=0.011), and the mean percentage changes in body weight of each group also positively correlated with the changes in ghrelin (r=0.740, *P*=0.000) (**Table 1**). According to Pasciak et al. (28), ghrelin-immunoreactive cell density was significantly lower in the treated groups (stomach fundus (13.5 ± 6.5) vs sham (34.8 ± 6.6), P<0.05), (body (11.2± 4.2) vs sham (19.8 ± 5.1),P<0.05), (duodenum (5.6 ± 2.2) vs sham (2.4± 0.9),P=0.081), and gastrin immunoreactive cell density in the stomach fundus was higher (76.2 ± 13.2 versus 65.7 ± 14.0, P=0.16). Paxton et al. (27) also discovered that ghrelin-immunoreactive mean cell density was significantly lower (15.3 versus 22.0, *P*<0.01), while fibrosis was increased in the gastric fundus of treated animals (*P*=0.07). These findings suggested that GAE reduces the number of gastrin-secreting cells in the gastric sinus with increased fundic fibrosis, leading to a decrease in ghrelin level.

#### Human Trials

Ghrelin was evaluated in three human trials involving obese patients (35, 37, 40). It was reported that ghrelin decreased most significantly within 1-6 months (*P*<0.05) but gradually recovered from 6-12 months (*P*>0.05), which was still lower than the baseline level. In addition, three trials examined other intestinal hormones besides ghrelin. At one and three months after surgery, Weiss et al. (38) reported GLP-1 and PYY levels elevated. Syed et al. (34) found decreased levels of cholecystokinin (CCK) and leptin after LGAE. Bai et al. (40) reported a similar decrease in leptin level. However, they did not perform a correlation analysis between weight loss and changes in gastrointestinal hormones (**Table 2**).

### Quality of Life

**Figure 5:**
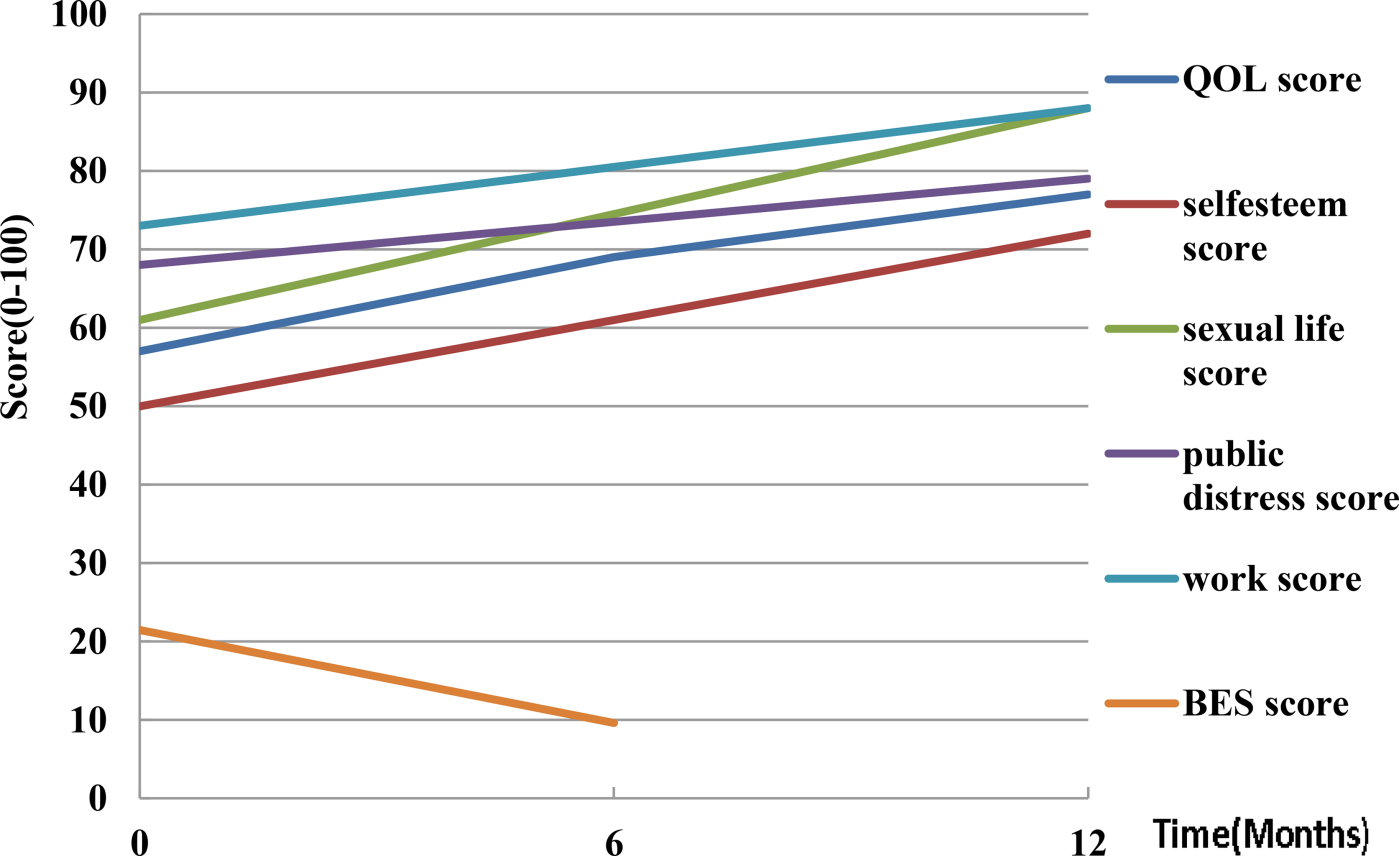
Quality of life changes post-LGAE in obese patients. LGAE, left gastric artery embolization; QOL, quality of life; BES, binge eating scale.

Five human trials assessed the quality of life (QOL). Weiss et al. (38) evaluated eating using the appetite and satiety questionnaire, and found that hunger and the 3-day food log decreased by 25.1 ± 27 points after three months. The second trial reported (33) that QOL scores increased from 57 ± 18 to 77 ± 18 (*P*<0.001) after 12 months. In addition, the mean physical function scores increased from 55 ± 18 to 70 ± 21 (*P*=0.007), self-esteem scores increased from 50 ± 30 to 72 ± 25 (*P*=0.011), sexual life scores increased from 61 ± 35 to 88 ± 25 (*P*=0.003), public distress scores increased from 68 ± 19 to 79 ± 19 (*P*=0.003), and work scores increased from 73 ± 17 to 88 ± 13 (*P*=0.007) based on the Short Form 36 (SF-36) questionnaire. Syed et al. (37) found a mean improvement of 9.5 points on the physical component scores and 9.6 points on the mental using the SF-36 questionnaire. Elens et al. (47) reported a satisfaction score of 7.7 ± 1.6 on a scale of 0-10 at the 6-12 months follow-up, with 12.5% of the participants finding the procedure very painful and would not do it again. Levigard et al. (48) found that the QOL score improved from 59.64 ± 5.59 to 69.02 ± 11.97 (*P*<0.05), while binge eating scores (BES) reduced from 21.50 ± 8.89 to 9.60 ± 4.40 (*P*=0.01) (**Figure 5**). These findings demonstrated that postoperative patient quality questionnaire scores increased, but a small number of participants found the experiment process to be too painful.

### Inflammatory Factors

Chronic inflammatory response is a major cause of glucolipid metabolism disruption in obese patients. Bariatric surgery reduces many inflammatory mediators and cytokines associated with obesity. The levels of inflammatory mediators, such as interleukin (IL)-6 and tumor necrosis factor (TNF)-α were decreased after bariatric surgery (49–52). One animal trial (31) showed that serum IL-6 (220.45 ± 7.54pg/mL/-35.1% in LGAE group, 219.23 ± 7.22pg/mL/-33.3% in RGAE group, 219.23 ± 7.22pg/mL/-2% in sham group,131.23 ± 3.43pmol/L/+2.4% in healthy group) and TNF-α (551.23 ± 75.82 pmol/L/-49.3% in LGAE group, 561.11 ± 79.72pmol/L/-47.2% in RGAE group, 558.23 ± 78.82pmol/L/-1.3% in sham group, 224.22 ± 47.81pmol/L/+5.4% in healthy group) levels were lower in the LGAE and RGAE groups than in the sham-operated group, suggesting that the inflammatory syndrome response may be reduced. However, there was a lack of information on human studies.

## Discussion: Influencing Factors for Efficacy

### Surgical Methods

Although the animals, embolic agents, embolic sites, and follow-up times used in the animal studies were not identical, it was shown that GAE either prevented weight gain in developing animals or reduced weight in adult animals. We re-calculated all human experiments and found GAE caused greater weight loss in the early stages, with a trend towards weight recovery that was still lower than baseline level after 24 months in eleven prospective studies. GAE results in weight loss of no more than 20%, which is far from sufficient for severe obese patients, and perhaps GAE should only be used in patients with mild to moderate obesity, especially those who have failed in lifestyle intervention or pharmacological treatment. We consider that obesity is a chronic disease and may require several successive GAE to achieve remission or conventional metabolic surgery after a first embolization. Pirlet et al. (42) reported that one patient who left gastric angiogram showed recanalization of the distal gastric left artery vascular system, underwent a second embolization procedure 18 months later, suggesting that re-embolization is not recommended. If they receive conventional metabolic surgery after a first embolization, the use of the left gastric artery will reduce the vascularization of the right part of the cardia and could be a handicap for performing a gastric sleeve or a gastric bypass. So, we need to prevent and treat patients recovering from ischemic injury because the rebound of weight and ghrelin levels may be due to re-vascularization of the gastric fundus. In addition, weight regain occurred in classic bariatric surgery after two years, follow up time is insufficient, further studies with larger sample sizes and longer periods are required to obtain stronger evidence. Additionally, Liu et al. (31) comparing left and right gastric artery embolization found that LGAE resulted in greater weight loss but without statistical significance. Legner et al. (30) distinguished embolic site and found the weight gain of the group embolized at the cardia significantly lower than that of the group embolized at the great curvature of the stomach and the sham operation group in rats. Therefore, further comparative studies are needed concerning the specific embolization sites of GAE.

### Embolic Materials

Arepally et al. used liquid sclerosants (e. g., sodium morrhuate, bleomycin A5 hydrochloride, and lipiodol emulsion) to achieve distal occlusion of all fundal arteries in developing swine, a procedure termed “gastric artery chemical embolization” (GACE). They found that chemical embolization is not consistently effective in reducing weight. The embolization of this fluid embolization was difficult to pinpoint, which could explain the occurrence of gastric ulceration and necrosis. Solid embolic materials were more likely to promote weight change compared to liquid material. Then, particles were used, including coils, gelatin sponge, and polyvinyl alcohol (PVA) particles ranging from 100-1200µm. Larger PVA particles and microspheres that were biocompatible, as well as a highly compressible embolic agent, have been widely used (21). The size of the embolized particles is a critical factor. Plasma ghrelin levels were similar between GAE pigs and control pigs, regardless of microsphere size. Five ulcers in five pigs embolized by using smaller microspheres (100-300µm), and three ulcers in five pigs embolized by using larger microspheres (300-500µm) (53), it is suggested that large particles may be a more suitable embolic material for GAE. Furthermore, they used x-ray-visible embolic microspheres (XEMs) and an antireflux catheter to improve safety and efficacy of GAE (14, 54–56). Still, comparative studies on the source, size, and method of embolization of embolic materials are required to determine the optimal embolic material.

### Gastrointestinal Hormone Changes Post-GAE

**Figure 6:**
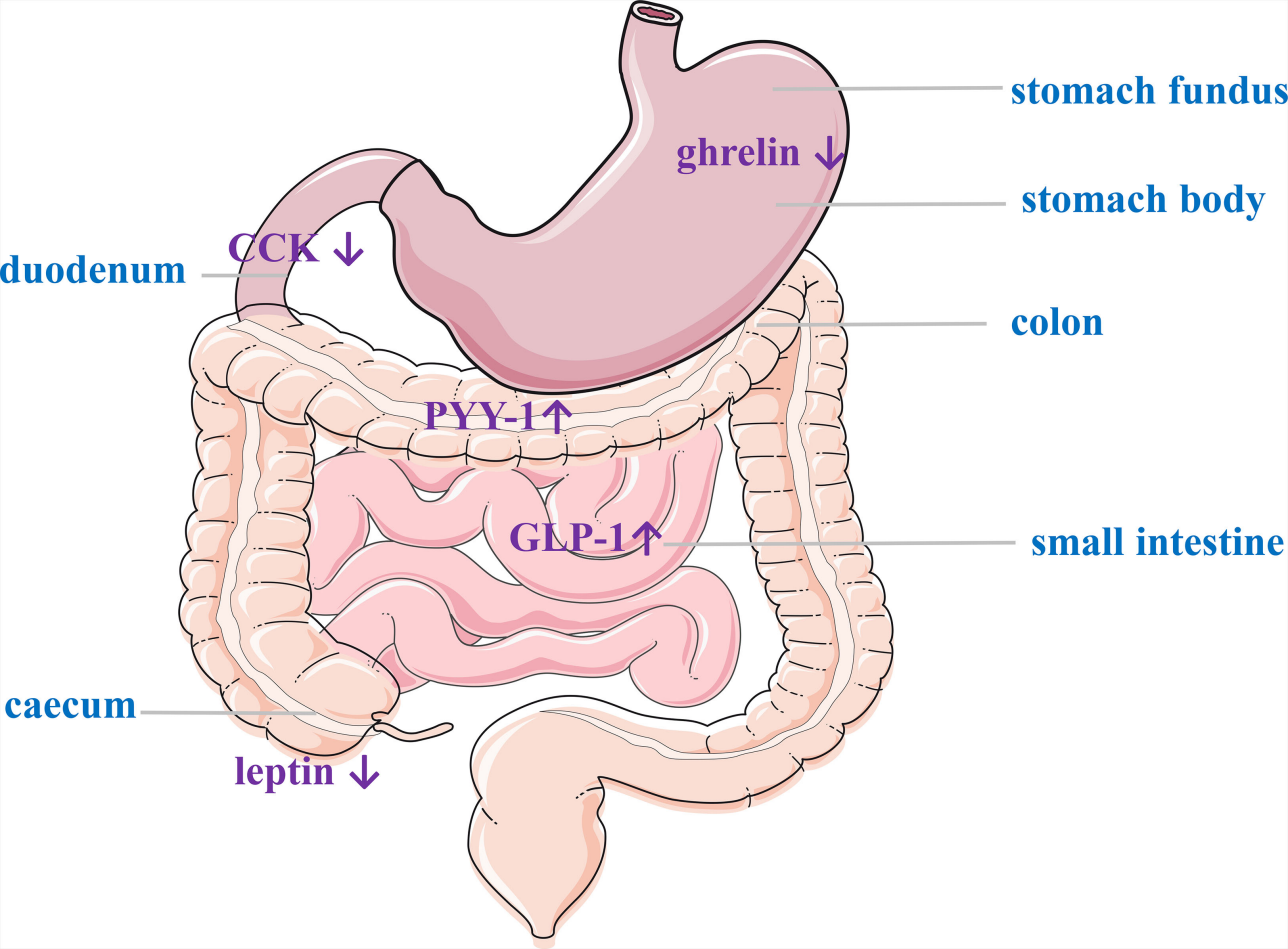
Gastrointestinal hormone changes post-LGAE. LGAE, left gastric artery embolization; CCK, cholecystokinin; GLP-1, glucagon-like peptide-1; PYY, peptide YY.

The mechanisms underlying bariatric surgery for the treatment of obesity and metabolic syndrome are not fully understood. An increasing number of studies have shown that many GI hormones levels are significantly changed after bariatric surgery, demonstrating that they may be important mediators that influence feeding behavior and regulate glucose level. The main GI hormones associated with energy homeostasis are PYY, GLP-1, CCK, OXM, gastrin, glicentin, and pancreatic precipitin (53). Both RYGB and SG lead to the postprandial secretion of GLP-1, PYY, and OXM, which can increase satiety (14, 54–56), these hormones regulate glucose homeostasis by altering insulin secretion and sensitivity during the early postoperative period (57, 58). Therefore, does GAE also result in weight loss by altering GI hormones? (**Figure 6**).

#### Ghrelin

Ghrelin is a 28 amino acid peptide secreted primarily from the fundus of the stomach (59) and in small amounts from the duodenum, pancreas, ovaries, adrenal cortex, and brain (60). It may increase appetite and lead to weight gain, and it’s the only gastrointestinal hormone that stimulates appetite. Many studies have reported that ghrelin level decrease after metabolic surgery, receptors in the arcuate nucleus (hypothalamus) bind and integrate signals sent to the satiety center (located in the nucleus solitarius), reducing hunger and appetite (61–63). Ghrelin levels were found to be decreased *in vivo* in nine animal and four human studies. But there may be some confounding factors in the results. On the one hand, circulating ghrelin consists of two forms: inactive ghrelin (90%) and active acylated ghrelin (10%) (64), none of the published studies have examined the active form (65) because the active form of ghrelin is unstable at a normal temperature (66). On the other hand, the lack of standardization in the measurement of ghrelin level in terms of sample collection schedule, collection method, follow-up period, storage of sample, and radioimmunoassay may affect the precision of the results.

#### Other Hormones

GLP-1 is produced by L-cells in the distal small intestine and colon in response to food intake and is considered the “ileal brake” on food intake. It provides satiety by activating receptors in the vagus nerve, proximal gastrointestinal tract, pancreas, brainstem, and hypothalamus. There is a wide range of GLP-1 secretion effects, including delayed gastric emptying, increased insulin secretion, and decrease gastric acid and glucagon secretion (67). PYY is mainly secreted by L-cells in the ileum and colon in response to food intake and, to a lesser extent, in the delta-, PP-, and alpha-cells of the pancreatic islets. The biggest change in intestinal hormone secretion after RYGP or SG is the significant increase in peripheral GLP-1and PYY in the days following surgery (68–71). Weiss et al. (38) revealed that there was an increase in GLP-1 and PYY after LGAE, suggesting that LGAE may cause changes in the levels of both of these gastrointestinal hormones associated with weight loss. CCK in the intestinal mucosa has the highest density of I-cells in the duodenum and proximal jejunum (72). The ingestion of protein, amino acids, and digested fat results in the maximal release of CCK (73). CCK promotes satiety by delaying gastric emptying through CCK receptors (stimulating pancreatic enzyme secretion and gallbladder wall contraction). The levels of CCK were elevated after RYGB (57, 74), with a gradual increase in secretion one week after SG (75). The effect of bariatric surgery on CCK homeostasis remains unclear. Similarly, only one trial (37) demonstrated a decrease in CCK after GAE. Leptin is released by subcutaneous white adipose tissue (WAT) (76) that acts as an endocrine signal by reducing appetite, activating pre-opioid melanocortin (POMC)-expressing neurons, and inhibiting hypothalamic agouti-related protein (AgRP) and neuropeptide Y (NPY). In addition, leptin increases the oxidation and absorption of glucose and free fatty acid in skeletal muscles, as well as promotes intrahepatic lipid reduction through fatty acid oxidation (77). The changes in leptin levels after GAE are inconsistent (30–32, 37, 40), and its relationship with weight loss has not been investigated.

Therefore, both animal and human studies have shown a decrease in ghrelin release from gastric fundic cells and an increase in GLP-1 and PYY which associated with reduced hunger and weight loss, and there was a positive correlation between weight loss and ghrelin, suggesting GAE may induce weight loss by lowering ghrelin. Yardimci et al. (32) found that SG can lead to more significant weight loss by altering the anatomy of the gastrointestinal tract compared with GAE although ghrelin decrease similarly in the two procedures. This suggests that GAE is not as effective as conventional metabolic surgery, with the latter causing the most significant weight loss due to alterations in the anatomy of the gastrointestinal tract, with changes in gastrointestinal hormone levels being a secondary factor. One previous meta-analysis (21) although demonstrated a significant reduction in MD of ghrelin levels among two animal studies (27–29) (MD: − 756.56, 95% CI − 1098.78, − 414.33, P < 0.001), but Hedges’ g analysis among three human studies (38, 40, 45) showed that GAE had not significantly affected serum ghrelin level (Hedges’ g statistic: − 0.91, 95% CI − 1.83, 0.01, P = 0.05). We have summarized the reasons for the different statistical results obtained. Firstly, ghrelin increased at 1 month, decreased most at 3 months, recovered slightly at 6 to 9 months, so, the time points for analysis were different. Secondly, we observed a decrease in ghrelin level in 9 animal experiments, with some statistics showing P>0.05 and some P<0.05. We did further correlation analysis, and discovered weight loss positively correlates with ghrelin which indicated that the left gastric artery may be reduce serum ghrelin and induce weight loss after LGAE. But, researchers need to establish a large number of randomized controlled trial (RCT) studies with the same subjects and compare previous metabolic surgery with GAE to complete a correlation analysis between weight loss and hormonal change to confirm whether gastrointestinal hormone change is an important factor in weight loss.

### Effects of Feeding Behavior

Decreased appetite is an important factor in weight loss, but whether appetite change is associated with gastrointestinal hormone change or with stomach ischemic injury is not clear. Previous studies didn’t record eating, so the structure and volume of diet require further investigation to accurately assess the effect of GAE surgery on feeding behavior and the efficacy of the latter on weight loss.

## Adverse Reactions

One pig that received the highest dose (2000 μg) during GAE died of ruptured gastric ulcers on postoperative day 1. Another pig died of peritonitis due to a surgical technical error that involved gastric fluid leakage from a poorly sealed biopsy site on postoperative day 2. The remaining adverse effects mainly included superficial gastric ulcer (12.5-100%), which was examined by endoscopy in six animal studies, embolization recanalization, and an increased vascular network at the cardia. Furthermore, the main adverse effects in eight human studies were gastric ulcers (10.5-60%) and superficial gastritis (25%), while a few patients had mild subclinical pancreatitis (5-20%) and epigastric discomfort (70%). However, the clinical symptoms of all patients were alleviated after being given proton pump inhibitors (PPI) one week before and one month after surgery (**Tables 1**, **2**). This indicates that the incidence of postoperative GI adverse reactions is mild and can be alleviated by perioperative administration of PPI.

## Conclusions

GAE is a new, innovative, image-guided approach for the treatment of obesity. Compared to invasive procedures, it is less harmful and its most common adverse effects are gastric ulcers, mild pancreatitis, and uncomfortable clinical symptoms, which can be alleviated by PPI preparations. It may be useful for mild to moderate obese patients who are struggling with a lifestyle-based weight loss program (78). However, there are still many issues with GAE that need to be addressed. Further RCT studies with large samples are required to compare the effects of different types of GAE to identify the best operation method and the optimal embolic agent, to determine whether GAE reduce serum ghrelin and induce weight loss, to compare the long-term effectiveness, safety, and health economics of GAE to classic bariatric surgery.

## Author Contributions

All authors listed have made a substantial, direct, and intellectual contribution to the work and approved it for publication.

## Funding

1.3.5 project for disciplines of excellence, West China Hospital, Sichuan University (No. ZYGD 18017).

## Conflict of Interest

The authors declare that the research was conducted in the absence of any commercial or financial relationships that could be construed as a potential conflict of interest.

## Publisher’s Note

All claims expressed in this article are solely those of the authors and do not necessarily represent those of their affiliated organizations, or those of the publisher, the editors and the reviewers. Any product that may be evaluated in this article, or claim that may be made by its manufacturer, is not guaranteed or endorsed by the publisher.
